# Intelligence, Correspondence, &c.

**Published:** 1823-06-01

**Authors:** 


### INTELLIGENCE.

### *Notice of the Anatomical Preparations of Harvey, lately presented to the Royal College of Physicians.*

They are six in number, and consist of the arteries, veins, and nerves of the body, displayed upon boards, to which they are attached either by some glutinous matter, or possibly, being dried upon the wood, adhere by the animal gelatinous substance itself. The blood-vessels are not injected, but the dissection of the cutaneous nerves, and of the ramifications of the arteries, is carried to great minuteness. If we consider that they are at least 200 years old, and only reflect for a moment upon the purposes to which they served, being beyond doubt the very preparations which the immortal Harvey employed in his lectures at the College, when he demonstrated the great discovery he had made, it is impossible not to consider them as objects of the most curious and interesting nature.

The history of their preservation, and the manner in which they have recently come into the possession of the College, adds, if possible, to their own intrinsic value. It is well known that Harvey had no children, but having endowed the College with his library and other splendid gifts, he left by his will the greater part of his property to his brother, whose daughter was married to the Lord Chancheller Nottingham, an ancestor of the present Earl of Winchilsea. These anatomical preparations remained at Burleigh on the Hill, the seat of that nobleman, who, a few weeks ago, presented them, through the President, to the College, accompanied by the following letter:—

*Copy of the Earl of Winchilsea's Letter to the President of the Royal College of Physicians.*

Sir, South Street, Feb. 22, 1823.

I have in my possession some anatomical preparations which belonged to the late Dr. Harvey, which I have great pleasure in offering, through you, to the College of Physicians, in the hope that they will consider them as worthy of their acceptance, and thinking that these specimens of his scientific researches can be no where so well placed as in the hands of that learned body of which he was so distinguished a member. I have the honor to be, Sir,

Your most obedient, humble Servant,

To Sir Henry Halford, Bart. WINCHILSEA.

President of the Royal College of Physicians.

*Copy of the Answer of the President, &c.*

My Lord,—I am desired by the Fellows of the Royal Coll. of Physicians assembled to make their most respectful acknowledgments to your Lordship, and to express their thanks, in the strongest terms, for one of the most gratifying and valuable presents which the College has ever received.

They trace, my Lord, in these interesting remains, the first steps by which physic was elevated to the dignity of a science, and though experience has made great improvements in the art of preserving such curious and instructive objects; yet, viewed as specimens of the earliest anatomical preparations ever made, to illustrate one of the most important discoveries ever disclosed to mankind for its benefit, by the great master himself, who first expounded the circulation of the blood, these relics are invaluable in the eyes of the College, and will be preserved, doubtless, to the latest period of their possible duration, with religious care. I am, my Lord, with most respectful attachment,

Your Lordship's faithful Servant,

HENRY HALFORD,

President of the Royal College of Physicians.

*College of Physicians, March* 24, 1823.

*To the Earl of Winchilsea, &c.*

### WIDOWS' FUND OF ARMY MEDICAL OFFICERS.

The third aniversary dinner of this most excellent institution was celebrated on the 16th of May at the Thatched House Tavern, Sir Everard Home, Bart. in the chair. We observed many of the most distinguished ornaments of the profession present at this festive scene, and several neat and appropriate speeches were made as the bumper toasts went round. The chaplain of the institution made two speeches—one in the form of a grace, which was very short and formal, before dinner, the other in form of thanks, which was long and witty, after the bottle had made its rounds. As an apology for the high church rather than the Presbyterian grace before dinner, he urged the fear that not only the guests but even the dishes on the table would have looked *cold* on him, if he had kept them half an hour blessing the "creatures" on the board before them. He thought it most natural, therefore, to be grateful *after* we had made sure of the favours sent for our use. When the president retired, (which was at a very early hour) Sir James M'Grigor was called to the chair by general acclamation, and numerous were the plaudits which greeted him as he took his seat. The song, the glee, the toast went round, and at 11 o'clock the company retired, after an evening of great hilarity and good humour

---

### PENITENTIARY, MILBANK.

In a former part of this number, we have slightly adverted to the nature of the sickness prevailing among the convicts at the General Penitentiary, Milbank. The subject has since excited much public interest, and considerable discussion, both on account of the novelty (in London) and fatality of the disease. The appearance of "sea scurvy," in all its protean forms, among the inhabitants of Terra Firma, after it has been banished from its native element by the intelligence of the medical officers of the Royal Navy, and the liberality of Government, together with the causes that have induced it, will necessarily form an interesting record of medical history, and we present to our readers such facts as we can glean from the authentic public documents already printed.

It will be remembered the Penitentiary was established, at the national expense, for the humane purpose of reforming young criminals, and those not hardened by a long course of vicious practices, and of returning them to society instructed in all the moral, religious, and useful duties of life. A committee of some members of the House of Commons, and others, superintends the government and management of the prison, which is, as usual, vested in a governor, matron, and subordinate prison officers. The prison contains about 850 convicts, and the medical department has consisted of a "principal medical superintendant," to be consulted on all necessary occasions, and a resident surgeon.

It appears that in March 1822, the attention of the superintending committee was directed to the reduction of the diet of the prisoners in health, which, at that time, consisted of the *daily* allowance of 20 ounces of bread, three ounces and a half of dressed meat, one pound of potatoes, about one pint of broth, and two pints of gruel or porridge, to each individual; and they requested Mr. Copland Hutchison's (the medical superintendant's) opinion "upon the reduction the above prison dietary is capable of, with a due regard to the health of the prisoners," which he gave in a letter of which the following are extracts.

"REPORT ON DIETARY TO THE COMMITTEE.

"SIR, *Leicester Square, 22d March,* 1822.

"The Committee having done me the honour by your letter of the 18th instant, to request my opinion upon the present Prison Dietary, and the

'reduction it is capable of, with a due regard to the health of the Prisoners,' I beg to submit the following observations, and scale of Dietary, for their consideration.

"The enquiry as to what reduction their present Dietary will admit, involves some serious considerations, and can perhaps only be finally determined by the results of experience. First,—It must not be sufficiently reduced, to impair the health of the prisoner, or render him unfit on emancipation to work for his own or his family's subsistence. This would be adding to the burden of the Parish to which the unfortunate man may belong, and inconsistent with that humanity, which tempers the justice of our laws. Secondly,—In considering this question, we cannot altogether take example from other Prisons or Houses of Correction; as, in the Penitentiary, the Convict is condemned to a much longer period of imprisonment, of which, being aware, his mind will have a much greater tendency to depression; and such exhaustion of animal spirits will be likely to terminate in disease; besides, it is obvious that a man may be very well able to subsist for a few months, a year, or possibly two years, upon a scanty allowance of food, without any apparent diminution of health or strength, but beyond this period, the powers of his constitution may not be able to sustain the trial. Thirdly,—The majority of the Prisoners sent to this Institution will, I believe, be found to belong to the Metropolis—to have been earlier initiated into crime—and to have been more successful in their nefarious pursuits, than those sent from the various County Jails throughout the Empire, and have in consequence lived higher as to eating and drinking than the other class; and therefore will be more subject to disease by any sudden transition from intemperance to a very small regulated diet.

"Considering then these circumstances, without adverting to others that might be urged; I should be disposed to give it as my opioion, that the Dietary for the Penitentiary should stand thus.

"The daily allowance of bread to be precisely what it now is.

"Gruel the same, night and morning, warm.

"One quart of broth to the males as *now** made; the same quantity to the females employed in the wash-house and laundry: to the other females one pint and a half only, during six days in the week.

"Eight ounces of meat of the *same kind as they have heretofore been accustomed to*,† on Sundays, with one pound of boiled potatoes, omitting broth on this day altogether, for all descriptions of Prisoners.

"Although this be my opinion, after much reflection, and some knowledge of the constitutions of convicts, during a period of nearly six years that I have been the principal medical officer of the Penitentiary, yet I consider the question as very difficult of accurate solution, and would suggest to the Committee to take the opinion of others in the profession who may have distinguished themselves in science—such as Dr. Wollaston, Sir Gilbert Blane, Dr. Baillie, Sir Everard Home, and Mr. Cline—that the best information may be obtained on this highly important and interesting subject; and if the Committee do think proper to come to such a decision, I should recommend that the Gentlemen fixed upon be requested, in the first instance, to inspect the existing state of health of the whole convicts.

"I am, Sir,

"Your very obedient Servant,

"*R. Auld, Esq.* "A. Copland Hutchison,

"*Sec. to the General Penitentiary.* "*Medical Superintendant.*"

The proposed reduction of Mr. C. Hutchison did not square with the Pythagorean notions of the committee, who, on the 4th of July, established the following scheme of diet:—

---

* *Six ounces of fresh meat to the allowance of each individual's broth.*

† *After being boiled, and without bone.*

(PER WEEK.)

"9 pounds 3 ounces of bread } or to be changed by one pound of potatoes for each half pound of bread. } *Farinaceous Food.*

6½ pints of gruel . . . .

12½ pints of broth (improved as before.)

"Animal food allowed to make the broth.—two ox-heads for each 220 persons; and it is ascertained that each ox-head produces of clear animal food 8 pounds on an average, but allowing another pound for animal matter contained in the bones and extracted by boiling—this gives 288 ounces among 220 persons (one ounce and one-third to each person) being a weekly allowance to each couple of nearly 19 ounces of undressed animal food, or *two ounces and two-thirds to each couple daily*;"

by which they entirely excluded animal food in substance from the diet of the convicts. The committee in their printed report state that this scheme of diet was appointed under the sanction of the "*best medical opinion*;" the author of this opinion is at present unknown, but is certainly not one of the professional gentlemen recommended to be consulted by their medical superintendant—it is fair to suppose, the author of the best medical opinion did not remember it was at variance with the facts and opinion of Dr. W. Philip, who found the most concentrated decoction of beef did not afford much nourishment.—*On Indigestion, p.* 134-5.

From the 4th of July the dietary of the committee was continued; and early in February last, four or five cases of the common forms of sea-scurvy occurred, which, on the 8th, was announced to the committee by the medical superintendant. Since that period the cases have been numerous, and have displayed the common symptoms of spongy gums in a few cases, of ecchymosis and a board-like hardness of the extremities, contraction of the hams, the goose-skin, &c. as well as the more rare ones, such as scorbutic dysentery, diarrhœa, &c. We are informed 27 have died in the Penitentiary since January, chiefly of scurvy in its various forms, and that there are, at present, about 200 cases of scurvy, scorbutic dysentery, and diarrhœa. Some post-mortem examinations have been made of those who have died of scorbutic dysentery, and there appeared the same ecchymosed state of the lower part of the alimentary canal that was evident in the legs of those who died of this complaint.

Soon after the scurvy appeared, the medical superintendant who possessed considerable experience in scurvy, was virtually superseded by two physicians, who, in their report, distinctly attribute the induction of this disease to the reduced diet, in which opinion Mr. Copland Hutchison concurs. As the subject is sub judice, we shall abstain from any remarks, and be contented with expressing a sincere regret that the "best medical opinion" should have proved the worst that could have been adopted, and that the committee have not paid a little more attention to the opinion and advice of their "medical superintendant."

---

Dr. Emerson continues his Lectures on Medical Botany, at the Argyle Rooms, and will continue them every spring. We have been much gratified with the manner and substance of these lectures, and conscientiously recommend them to the rising generation of students in the West end of the town.

---

*Stricture of the Œsophagus,** (retrospective.) We are of opinion that facts are independent of dates, and that valuable suggestions have been thrown out before we were born. We conceive it to be no useless occupation to try back occasionally, in order to rescue some of these facts and observations from the oblivion which TIME brings over all things. Dr. Munckley, Physician to Guy's Hospital, some fifty years ago, relates a case of stricture of the œsophagus, which bore all the phenomena of organic disease. The patient was about 40 years of age when admitted

* Dr. Munckley, Physician to Guy's Hospital, in the year 1767.—(Trans. of the College, vol. 1.)

into the hospital, and complained of inability to swallow food, even of the softest kind. "Whatever she attempted to swallow, after staying some time in the throat, was thrown up again, by what appeared, from her description, to be a kind of convulsive motion of the œsophagus." The complaint had been coming on for some years, but latterly had alarmingly increased. She was now much emaciated—her voice hoarse, and her breathing disturbed. She could indicate the seat of the obstruction, but nothing external could be perceived. Dr. M. placed the patient under a course of mercury, keeping up a gentle spitting for six weeks, in which time every symptom of the disease disappeared, and the patient was discharged cured. The author has selected this case merely as a specimen of his general practice, by which, he had reason to believe, he saved many lives. "The only medicine," says he, "from the use of which I have ever found any service in this complaint, is mercury; and in cases which are recent, and where the symptoms have not risen to any great height, small doses of mercury, given every night, and prevented by purgative medicines from affecting the mouth, have accomplished the cure. But where the complaint has been of long standing, and the symptom has come on of the food's being returned through the mouth, in the manner above described, a more powerful method of treatment becomes necessary. In this case I have never found any thing of the least avail in removing any of the symptoms, but mercury used in such a manner as to raise a gentle but constant spitting—and this method I have pursued with the happiest success." Our author does not pretend that this success was invariable. Some cases resisted the remedy, but many gave way under its influence. In such a dire affliction, and where we all know mechanical dilatation is too often impracticable, it may not be unwise to bear the above observations and facts in mind

---

*Popular Medicine.** We have not time or inclination to enter into the often litigated question respecting the propriety or impropriety—the policy or impolicy of treatises on medicine, addressed solely to the non-professional reader. Our own opinion decidedly is, that such works do injury to the public at large, and put money into the pockets of the regular practitioners. The well educated student can with difficulty avail himself of written descriptions and precepts, when he first enters on the practice of his profession—how then is it possible that the "clergy and heads of families" can apply this written knowledge to the treatment of diseases, the real nature of which requires all the discrimination of the physician to investigate? The public, however, will never be convinced of their own incapacity for treating diseases till the domestic medicine chest fails, and therefore works of the description now before us will continue to be written and read per omnia secula seculorum. All that the regular medical practitioner can do, then, is to advise his patient (if he will have a popular treatise) to purchase that one which is least likely to lead him astray, and which evinces most knowledge of the subject. While we venture to say that Dr. Thomas has not added to his reputation by the drudgery of compiling a system of domestic medicine, we have no hesitation in asserting that the present work is far superior to all those now in the hands of popular readers. This assuredly is no great praise, considering the wretched compilations which have, for some years, misled the public; but still we think it our duty to make this statement, in order that the professional public may know what to recommend in the few cases where their opinions are asked.

---

† The Way to preserve good Health, invigorate a delicate Constitution, and attain an advanced Age; together with a Treatise on Domestic Medicine, &c. By ROBERT THOMAS, M.D. &c. Octavo, pp. 705. Lond. 1822.

